# Programmatic Support for Peer Specialists that Serve Transition Age Youth Living with Serious Mental Illness: Perspectives of Program Managers from Two Southern California Counties

**DOI:** 10.1007/s10597-023-01136-8

**Published:** 2023-06-15

**Authors:** Christopher Magana, Todd P. Gilmer, Michelle R. Munson, Nev Jones, Jose Luis Burgos, Victoria D. Ojeda

**Affiliations:** 1 Herbert Wertheim School of Public Health, University of California San Diego , 9500 Gilman Drive MC 0725, La Jolla, California, 92093-0725 CA USA; 2 Silver School of Social Work, New York University, New York, NY USA; 3 School of Social Work, University of Pittsburgh, Pittsburgh, PA USA

**Keywords:** Peer Specialists, Peer providers, Professional development, Transition age youth, Serious mental illness, Public mental health services

## Abstract

Peer Specialists (PS) often work in outpatient mental health programs serving transition age youth (TAY). This study examines program managers’ perspectives on efforts to strengthen PS’ professional development. In 2019, we interviewed program managers (n = 11) from two Southern California Counties employed by public outpatient mental health programs (n = 8) serving TAY and conducted thematic analyses. We present themes and illustrative quotes. PS’ roles are highly flexible; thus, PM support PS to strengthen skills to address organization-facing and client-facing responsibilities. PM addressed time management, documentation, PS integration into the organization, and workplace relationships. Trainings to better support clients included addressing cultural competency to serve LGBTQ TAY and racial/ethnic subgroups. Diverse supervision modalities address PS’ diverse needs. Supporting PS’ technical and administrative skills (e.g., planning, interpersonal communication skills) may aid their implementation of a complex role. Longitudinal research can examine the impact of organizational supports on PS’ job satisfaction, career trajectories, and TAY clients’ engagement with services.

## Introduction

The use of Peer Specialists (PS) by mental health service programs has grown considerably (Chinman et al., 2014; Shalaby & Agyapong, 2020). Although specific job titles and responsibilities of PS vary by program, these positions are frequently defined as being occupied by someone with personal experience of mental illness who draws on that experience to support others in their recovery. PS’ roles are diverse and may include leading individual support meetings, supporting group facilitation, mentoring clients, case management, and leading community outings (Ojeda, Jones, Munson, Berliant, & Gilmer, 2020). The use of PS is recognized by the United States Department of Health and Human Services as an evidenced-based mental health service model, and peer roles are widely accepted as a best practice by many mental health services programs, including those serving transition-age youth (TAY, youth ages 16–24) living with serious mental illness (SMI) (Mahlke, Krämer, Becker, & Bock, 2014; Simmons et al., 2020).

Compared to other adult populations, TAY have a relatively greater incidence of mental health challenges, in part due to the emergence of symptoms for many mental illnesses in early adulthood; historically, TAY have also been harder to engage and retain in mental health services than other adult groups (Bonnie, 2014; Cusick, Havlicek, & Courtney, 2012; Davis, 2003; Munson & McMillen, 2009; Vostanis, 2005). PS can play a critical role in the delivery of mental health and social support services for TAY with SMI and may help improve access to and continued engagement in mental health services by acting as a bridge between traditional service providers (e.g., therapists, psychiatrists, case managers) and TAY clients (Munson et al., 2021). Early engagement for the treatment of psychosis aims to reduce the duration of untreated illness, which is associated with improvements in clinical symptom severity and increases in rates of recovery (Hegelstad et al., 2012; Kane et al., 2016). A 2009 study of natural mentoring of youth transitioning out of foster care found that those with a mentor reported improved psychological outcomes (Munson & McMillen, 2009). More recently, an Australian qualitative study found that young adult peer workers (ages 17–21) believed that both their lived experience and youthfulness were critical to their ability to effectively support youth (Simmons et al., 2020). A recent study employing administrative mental health service utilization data in Southern California found that PS may also help to reduce racial health disparities by strengthening the engagement of minority TAY in mental health services (Ojeda et al., 2020).

Despite the growing use of PS within outpatient mental health service settings, less is known about how program managers train the peer workforce, support their work, and assist in their professional development (Ojeda et al., 2016; Ojeda et al., 2020). PS may benefit from diverse training strategies. For example, a 2020 national online survey of PS serving TAY found that 57% of respondents identified a training or skill need, including needing training to improve collaboration with other service providers, integrating technology in alignment with patient privacy policies and ethical principles, and meeting the specific needs of underserved populations including racial/ethnic minority TAY, and TAY with SMI and/or substance use disorders (SUD) (Jivanjee et al., 2020). Another large national survey of PS across the U.S. (N = 801) found that 60% reported having never had a single conversation with their supervisor about career development and advancement, and there were numerous other areas in which organizational support for career development was lacking (Jones, Teague, Wolf, & Rosen, 2020). Qualitative data from that study also detailed numerous perceived barriers to career development and advancement, including stigma and discrimination in the workplace and inadequate compensation (Jones, Kosyluk, Gius, Wolf, & Rosen, 2020). Importantly, a recent study which sampled both PS and their supervisors identified eight key factors for PS’ “on-the-job success”, including training, regular and individualized supervision, and skilled communication with colleagues (Delman & Klodnick, 2017).

This study reports qualitative findings from a sequential mixed-methods study investigating the role of peer support services in reducing disparities in the use of mental health care among TAY in two large, diverse California counties. This analysis draws on interviews with mental health program managers’ which sought their perspectives on strategies to support the professional development and capacity of PS. This study fills an important gap in the literature by addressing aspects of professional development needed to ensure PS’ success in the TAY-serving mental health services workforce.

## Materials and Methods

### Participants

We conducted a sequential mixed-methods study involving a quantitative survey in Phase 1 and qualitative interviews with program managers of publicly funded outpatient mental health programs in San Diego and Los Angeles Counties in Phase 2—these interviews are the focus of this analysis (Creswell & Hirose, 2019). Each county first provided the investigators with a list of county-funded mental health programs that serve TAY and in 2018-19, programs were queried to determine their use of PS (Ojeda et al., 2020). Next, we employed purposeful sampling to identify programs with 100 or more clients TAY clients, including Black and Latinx clients, and that also employed three or more paid peer providers; we identified eight unique programs to participate in qualitative interviews (n = 5 from Los Angeles County, n = 3 from San Diego County) (Palinkas et al., 2015). Between September and November 2019, program managers at these programs were contacted to participate in interviews and the principal investigator conducted interviews with 11 participants (n = 6 from Los Angeles County, n = 5 from San Diego County). Interviews ranged from 60 to 90 min and were digitally recorded.

### Ethical Approval

Ethical approval for this study was granted and by the Human Subjects Research Protections Program of the University of California San Diego (Protocol #171,748), the Los Angeles County Department of Mental Health (Protocol #332), and the San Diego County Department of Behavioral Health Services (no Protocol # provided). Participants provided written informed consent for their data to be used in research.

### Program Manager Interview Guide

The interview guide included semi-structured open-ended questions and probes that elicited program managers’ perspectives on peer specialist roles (see Tables 1 and 2), training needs (see Tables 3 and 4), and organizational strategies (see Tables 3 and 4) used to support PS professional development. Before initiating the interviews, the facilitator defined Peer Specialists’ services as “*services that are provided by peers—individuals who have experienced with mental illness and assist others in their recovery.”* TAY were also defined as youth and young adults between the ages of 16 and 24 but with the understanding that this definition may vary slightly across programs.

**Table 1:** Program managers’ descriptions of peer support specialists’ (PSS) characteristics

Theme			Subtheme			Exemplary quotes			
1. Connections to TAY clients are built on PSS’ lived experience and an authentic, non-judgmental personality				1a. Lived experience is key to the PSS role				*I would say the peer specialist role is utilizing that lived experience and being able to connect with consumers in a way that the more professional staff don’t really have that same experience, so the connection is a bit different because it’s ‘I’ve been there, I’ve done that, I’ve experienced it, I am a testimony to what recovery looks like, recovery is possible…’*	
				1b. PSS are authentic, friendly and communicative				…*They’re very, and I’m sure you saw, they’re very outgoing and warm and genuine and they’re very hard workers and it’s such an important role because they are able to relate to the client and sometime the clients will open up to the peer supports more or initially before they open up to even to the therapist… I mean I think all my peers are able to initiate conversations. You saw them. They’re not super shy. But they’re able to do so in a gentle way. So, they have good speaking skills…*	
				1c. PSS are empathetic and non-judgmental				…*a lot of it has to do with being empathetic toward others even though they have their own stories. Even though I’ve experienced something similar doesn’t mean that because you are experiencing what I’ve experienced, what I did to help myself is going to work for you. So, there’s that level of empathetic understanding how my experience applies and being able to communicate that…*	
2. PSS-client compatibility in demographic traits and interests can foster the PSS-client relationship				2a. PSS may share age or interests with clients				*I think when my clinicians are trying to link them to the four peer supports that I have, I think that temperament and demeanor is one, like is someone more shy, maybe they want someone more gentle. You know everyone has different personalities, but also a big thing is their interests. So, I know this client is really into anime and really into that, so I have this peer support who’s into that and likes art and linking them together. I have a client who’s really into sports and skateboarding and music and photography and I have a peer support who’s into that as well, so I think interest and temperament is probably one of them…*	
				2b. PSS may share race/ethnicity, culture, language with clients				*I think what a lot of it comes down to is our staff being culturally competent. I think we’ve gotten lucky that naturally, some of our staff have a similar background or makeup as the community does, so I think that makes it helpful for our TAY clients to engage in Services when they can see someone that’s maybe reflective of some of their similar backgrounds in terms of race ethnicity, that sort of thing.*	
3. PSS often have a unique, organic relationship with clients due to the flexible nature of the PSS role				3a. PSS work in diverse settings to support clients’ evolving needs				*I mean, you know meeting them [i.e., clients] where they’re at, taking them to the community, working on social skills, I mean that’s such an important part of the peer supports and getting to know them just out of the clinical space like an office. So, the therapist, I mean most of the therapy happens here in the office, they’re in an office, they’re doing therapy, they’re doing interventions. The peer supports take them out. Ride the bus with them, show them beautiful San Diego that maybe they haven’t seen before, take them to the zoo, take them to free museum day and really get to know them and teach them social skills which is a really big part of improving their functioning.*	
				3b. PSS have organic, less structured relationships with clients				…*I think peers are more organic in their approach and they don’t have these guidelines and models that they have to maintain fidelity to and may be able to be more flexible. Another thing is they have smaller caseloads where I feel like the mental health professionals with licenses, credentials, and degrees, have a far greater caseload where they aren’t able to do a lot of the connection work, a lot of the rapport-building that I think peer supporters, peer workers are able to do. …I do feel that there is this sense of wrapping it up a little more quickly for social workers to get to that next client, because they have that larger caseload and they need to continue to see that next client and the next client and I would say that the peer workers, Community workers don’t have that restraint.*	

**Table 2:** The role of peer support specialists (PSS) and the services they provide

Theme		Subtheme		Exemplary quotes
1. The PSS role is expansive and inclusive of all stages of service utilization		1a. PSS conduct outreach to engage youth		…*our TAY peer specialists go to outreach fairs or health fairs at the different schools and what that looks like is it’s a booth that we set up with a ton of other community providers and we provide information as to what our services are, specific to our population. We like to try to bring a snack and some sort of like fun enticing thing to draw them into the table…We do those outreach events. With some schools we do go out and do the screenings at school so that the clients don’t necessarily have to come here for the screening aspect of it. So, we screen them at the school when they’re at the school and they could come in and have an appointment with us. We do have a TAY specific intake appointment because we can understand that it can be challenging for our TAY population to be successful in our walk-in model of intakes, so we do have a TAY specific intake spot that we have every week.*
		1b. PSS increase client comfort in utilizing mental health services		*I think those that are new, that are new coming into mental health services really benefit from having the peer services. Seeking treatment for the first time no matter what age you are can be scary, so having an additional person to support, especially for our younger TAY clients can be really helpful and it can also be helpful because maybe they don’t have a lot of education surrounding their mental health. So, the peer can be really instrumental in helping to provide a psycho education to the TAY age range.*
2. PSS help meet clients’ basic needs and model recovery		2a. PSS addressing clients’ basic needs		Transportation …*If the participant needs help taking the bus, public transportation, they will do training…, if they’re needing assistance with the use of public transportation, if we just feel like they would benefit from the peer support specialist service, a PSS will be assigned to that team…* Housing …*I want to think also about another consumer who received a lot of support when it came to getting housing. And she had had a baby and was trying to get affordable housing and I think that was very impactful for her, she was intimidated by the whole application process, looking for housing, going on these interviews, and just having that peer support all the way through every step of the way was very significant for her and she did end up getting that housing…* Linkages to Medical Care *…I see a lot of how impactful peer Support services are at the wellness center. I feel like we really get to do a lot of it there, have a larger team of community workers and they’re working one-on-one, just the phone calls. It’s remarkable. They have these connections and fear of going to the dentist, connecting them with resources for dental work and having a primary care physician, so many consumers have fears of health care services and that’ll be a big barrier with socializing is the how they feel about their teeth and how they look and getting them to the dentist. That goes back to what I said about them having that passion, the community worker being passionate about helping that client overcome the barrier…*
		2b.PSS model recovery and practice independent living skills		…*If the participant recently moved into their own apartment, the peer will help model those skills whether its hygiene or taking them shopping for clothes, cleaning the apartment, these are all things that our peer support specialists do…If the peer support specialist is helping the client budget, they too should have the ability, you know, that’s one of the ILS [independent living skills] that they too should have the… you know, hygiene, you have to look presentable when you go out to a job. So definitely communication, you have to have effective communication skills and ILS…They do take them to shopping, because we provide Flex funds for the clients to provide, for whatever they need, hygiene, clothes…*
		2c.PSS lead social activities		…*So, we have someone who is a musician back there and you’ll meet him today and so knowing that we are starting a music program. So, we bought a bunch of guitars and a drum set and he’s running music groups and he’s engaging youth in learning how to play the guitar and talking about music and all this stuff. So that’s just a specialty thing for him and it’s done a ton of engagement for the youth. We have a staff who’s into art and has worked with kids and starting our groups. We have a staff that just likes doing puzzles and she’s asked us to order a butt load of puzzle, so I’ve got closets full of puzzles and she’ll just bring out a puzzle, throw it out on the table, and by the end of the day she’s sitting there working on a puzzle surrounded by kids…*

**Table 3:** Program managers’ support for peer support specialists’ (PSS) organization-facing responsibilities

Theme	Exemplary quotes
1. PSS can benefit from trainings to improve time management and documentation practices	…*I tell candidates that the biggest challenge for any staff coming into this program, and I’m sure for other mental health program as well, the challenge is finding the balance between providing good client care service and documenting. Everybody wants to run out there and serve clients, but you need the paperwork to justify, that way we can continue to get the funding to provide the services. So, I train our staff to look at it not as two separate pieces but as one, that providing good client care service also includes billing and documenting the client’s story. Yeah, so that is a challenge for all staff even for our peers, our peer support specialists…*
2. Supporting PSS in their workplace relationships is an important component of organizational support for PSS	*So, our program is a team-based approach so definitely I’ve been trying to get everyone to come from that approach and to work at that and to collaborate with one another if you need something else. Especially with my TAY peers, is something I’ve been working on diligently because their schedule is a little different from the rest of my staff. So, I’ve made sure to switch our staff meeting so they’re present, …so now they’ve been incorporated into our staff meetings and I’m constantly trying to reinforce that there’s more people to make sure that they socialize and collaborate and they communicate and they explore the different positions and learn from one another of what each one has to offer.*

**Table 4:** Managers’ support for peer support specialists’ (PSS) client-facing responsibilities

Theme		Subtheme		Exemplary quotes
1. PSS need trainings across diverse areas to support clients		1a. Mental health trainings (e.g., CBT, Trauma-Informed Care, WRAP, Crisis / De-escalation)		*I mean the safety training is really important, but I would like more trauma-informed care training. …I want my staff to be really comfortable talking to youth about what they’ve experienced. I’ve heard from some, not just my peers but from my other staff like oh I don’t want to open that box because I’m not qualified to navigate with them through the trauma and what if they start talking about it and you know, I’m not able to help them. So, I really want my whole team to feel more confident about that. So, I’m looking for trauma-informed care training.*
		1b. Cultural competency trainings for racial/ethnic subgroup		…*Other trainings, you know cultural competence is always a huge one. I get creative in kind of taking from my team what do they want to learn? We had Trans Family Services come in and do a transgender training, we had LGBTQ from our safe place come in, we’ve had Middle Eastern Services come in and how to work with that population. We had Indian Health Council and working with the Native American population, so I love cultural competence and it’s really important.*
		1c. Training for appropriate self-disclosure		*Something that we tell our peer specialists they don’t necessarily have to share their story. They’re trained individuals, they know what they’re doing. It depends on their judgement. If it will benefit the client or if it will not benefit the client, then they just keep it to themselves. … we don’t ever want them to feel like they have to tell their story, they can use aspects of their story as they feel comfortable to connect but part of the training that they go through is about boundaries, what’s appropriate to share with clients, what’s not appropriate to share, that’s sort of thing. They do a lot of that in the training that’s provided.*
2. Cultural competency to serve LGBTQ community is important for PSS services		2a. Leveraging community partnerships to build PSS’ cultural competency to support LGBTQ clients		*…throughout our clinic, we have flags, we have signs about it being a safe environment, you know, the bathrooms have been changed to gender-neutral signs. So, we’ve done a lot with our infrastructure to make it a safe space. We’ve had many all-staff and services from our local specialty agencies like the center in Long Beach, they’ve come over. Exodus actually has a specialized program, so we’ve had them come over to do presentations for us on just the LGBTQI population because it’s all changing and adding more letters to it. So, we just had one recently, another one that was really, really cool and then I have a couple staff who are super interested in treating that population. What they’ve actually done is created a support group and it takes place here every week, they had it yesterday actually. They have it on Tuesdays. And it’s an open group and it’s just sort of like a drop-in if you want to come in, you can come in. So, it’s kind of cool to see, they started off with like one person and now it’s grown and grown. And we also had a pride event this year and that was really well-attended, and it was a big event. We had speakers, we have food, so that was really cool too. That was the first in our service area that any of our directly operated programs that had a pride event. We’re really trying to engage the LGBTQ population.*
3. PSS benefit from multiple types of supervision		3a. Individual Meetings		…*So, of the team lead, all of them are therapists. They work with the peer support specialist, whether it’s coaching, whether it’s training, whether it’s one on one, identifying challenges. So, they meet with their team lead at least two times a month to review the productivity, to get feedback, there’s real-time feedback whether it’s from myself or any of the management team. I’ll sit with them, I’ll help them if they’re struggling with documentation or meeting productivity and deadlines, I’ll meet with them. I’ll create action plans to help them be successful in whatever their goal is in the workplace.*
		3b. Group Meetings		*So, I lead the peer support supervision. Once a month, I meet in here with a peer support team. I might have some agenda items that I want to go over and then I’ll write on the board what do you want to come over today. And they might be like I had this safety concern or can we… because sometimes I have to do admin stuff like do, we have balance caseloads, does your caseload of 25 and this person has a caseload of 8, like what needs to happen. So, I have to do the admin stuff, but I also open it to them like what do you want to talk about today…*

### Data Management and Analysis

The investigators used MaxQDA 2020 software (VERBI Software, 20,219) to facilitate thematic analysis, as described below (Clarke & Braun, 2014; Maguire & Delahunt, 2017). First, interview audio files were transcribed verbatim by research assistants. Next, a coding scheme based on topics identified in the interview guide was developed and reviewed by the co-authors (CM, JLB, VO) for accuracy. The codes were applied to two transcripts by two co-authors (CM, JLB) who also used open coding was also used to identify emergent codes. Following the coders’ consensus, the coding scheme was finalized and applied to two new transcripts by both coders to assess inter-rater reliability. After resolution of coding conflicts and minor revisions to the coding scheme, satisfactory inter-rater reliability was achieved (i.e., 70%). The final code list was applied to the remaining transcripts by a single coder (Landis & Koch, 1977; O’Connor & Joffe, 2020; Roberts, Dowell, & Nie, 2019). Coded text segments across all interviews were collated into code reports and the co-authors (CM, JLB) summarized the code reports while assessing for similarities or differences in content and illustrative quotes were selected for inclusion in thematic data tables. To contextualize how programs support PS in their service delivery and professional development, we first describe how program managers perceive the peer role and characteristics of those successful in this position followed by program managers’ strategies to support PS who serve TAY clients.

## Results

### Characteristics of Peer Specialists

Program managers were asked to characterize the Peer Specialist role (PS) and how these staff are unique from other staff members (see Table 1, Theme 1, subthemes 1a, 1b, 1c). The emergent theme revealed that connections to TAY clients are built on PS’ lived experience and having an authentic, non-judgmental personality. Managers consistently identified lived experience with mental health disorders (e.g., personal experience, or with a family member or other close contact) as a job requirement. Managers felt that those who excel in the PS role are authentic, friendly, and empathetic– traits that were perceived to be related to their lived experience. Managers conveyed that their PS colleagues often displayed a passion for service and have strong communication skills which enable them to share their experiences in an empathetic, genuine, non-judgmental way. Finally, managers characterized their PS as friendly and approachable; they felt these traits help clients feel welcome and support service use.

Managers were asked to identify any characteristics for which matching between PS and their TAY clients was important (see Table 1, Theme 2, subthemes 2a, 2b). The data suggest that compatibility in demographic traits and shared interests can help the PS-client relationship. For example, several managers noted that having PS that are similar in age to TAY clients seems to help the PS relate to clients due to a shared culture of youthfulness. Similarly, managers identified mutual interests (e.g., music, photography, sports, art) as a means for peers and clients to build a rapport. Managers’ responses varied regarding the importance of matching based on race/ethnicity. Several noted that racial/ethnic or linguistic concordance (e.g., fluency in/ability to speak Spanish) can support TAY clients’ engagement in services; this could be achieved by hiring peers who share characteristics of racial/ethnic subgroups.

Managers were asked to describe ways in which the PS role compared to that of other mental health service providers (see Tables 1, Theme 3, subthemes 3a, 3b). Our analysis indicated that PS have a unique, organic relationship with clients due to the flexible nature of the PS role. For example, several managers stated that PS engage with clients in diverse settings, including on-site as well as in the community, including in clients’ homes; in contrast, psychiatrists’, therapists’, and other staff members’ interactions with clients were typically limited to programmatic settings. Managers also frequently characterized PS’ work as flexible and less structured than that of other staff: for example PS are more likely to have informal meetings vs. structured sessions with clients. The relationship between PS and clients was also described as “more organic” and it may evolve over time due to clients’ changing needs. In contrast, non-PS staff may go “by the book”, adhering to their professional boundaries as outlined by their discipline’s best practices.

### Functions of Peer Specialists

Table 2 presents managers’ descriptions of the PS role, which is expansive and inclusive of all stages of service utilization. Managers reported that PS often conduct outreach to engage TAY in mental health services (see Table 2, Theme 1, Subthemes 1a, 1b). For example, PS might attend health fairs, host educational activities in the community or conduct mental health screenings at schools. PS were described as effective and instrumental in connecting underserved clients (e.g., clients unfamiliar with or intimidated by mental health care, unsheltered youth) to mental health services, often for the first time, or supporting their continued engagement with mental health as well as other services. Managers felt that PS’ disclosure of their lived experience helps build rapport and trust with clients and enables PS to address TAY clients’ concerns, translate mental health jargon into lay language, and create a welcoming environment in mental health program venues. From managers’ perspectives, PS tend to use their lived experience to model recovery for clients and increase clients’ comfort with utilizing mental health services. PS are also important in helping address TAY clients’ basic needs (see Table 2, Subthemes 2a, 2b, 2c). Notably, across all organizations, managers explained that PS must be flexible and adaptable, performing tasks across numerous domains to meet clients’ diverse needs including securing or providing transportation or housing, and facilitating linkages to medical care, modeling and practicing independent living skills (e.g., use of public transportation, shopping, hygiene, housekeeping, budgeting), and leading groups and or other activities (e.g., music/art events) in support of the recovery process.

### Program Support for Peers’ Organization-Facing Responsibilities

Program managers described ways in which their programs support PS to carry out their administrative responsibilities (see Table 3). Managers frequently discussed the importance of documentation by PS, both in the service of clients’ care and for continued financing of services (see Table 3, Theme 1). However, they also identified challenges PS and other staff may have with documentation, including determining what information to record or carving out the time to fully document their work. In response, many managers described leading discussions regarding the critical role that documentation plays in maintaining quality care. Managers also sought out or provided documentation-focused technical trainings (e.g., what to document, how to prepare notes in order to meet reimbursement standards). Some managers carried out ongoing check-ins with PS and provided regular feedback on record-keeping. Programs also addressed time management challenges as they pertained to balancing documentation and client services. Thus, several programs hosted “admin parties” where PS could catch up on paperwork together and receive help from their supervisors if needed. PS received mentorship on time management, particularly as it pertained to conducting client services out in the field; one program required notes to be prepared within a pre-determined period (e.g., 5 days) and PS received devices (e.g., cell-enabled tablet computers) to support documentation while in the field and the information was fresh.

Managers reported supporting PS as equal and valued team members (see Table 3, Theme 2). Specifically, some made a conscientious effort to ensure that PS were present at programmatic and client-care meetings; this was identified as a potential challenge given PS varied schedules as well as service delivery model which resulted in PS engaging with clients in the community. Some managers sought to create non-hierarchical teams which valued and respected all team members’ insights as these could support PS’ participation in team discussions for the benefit of client care; this was identified as a need given that health care is often hierarchical. Some managers felt that supporting enhanced communication between PS and other staff members could lead to a greater understanding of PS’ contribution to client engagement in services and the recovery process. Other methods to involve PS included having PS host team building exercises and lead staff meetings. One manager also suggested that holding trainings to explain the duties and roles of the different team members, including the unique role of PS, would benefit all staff members. Finally, managers also identified a need to support the professional development of PS by helping them navigate difficult conversations, work-place disagreements, and determining the sharing of personal information for the benefit of the client.

### Program Support for Peers’ Client-Facing Responsibilities

Table 4 presents managers’ descriptions of how programs support PS across diverse areas to support client-facing work. Managers felt that certain mental health trainings were essential for preparing PS to ensure client safety and well-being (e.g., Wellness Recovery Action Plan [WRAP], trauma-informed care, Seeking Safety, identifying and responding to suicidal ideation) (see Table 4, Subtheme 1a). Cultural competency training was important for serving diverse racial/ethnic groups and sexual and gender minority communities; importantly, these trainings were often provided by community partner agencies with expertise in sexual and gender minority needs and services (see Table 4, Subtheme 1b, Theme 2, Subtheme 2a). Additionally, programs often formed partnerships with lesbian, gay, bisexual, transgender, and queer (LGBTQ)-focused organizations with the goal of ensuring that PS and other staff were aware of the range of resources available from community partners. Managers reported that these collaborations and greater agency awareness of LGBTQ issues also benefitted the program more broadly by helping create a physically welcoming space for LGBTQ TAY. Additionally, managers reported that training PS on self-disclosure and how to use their experiences in supporting clients is especially helpful when PS are early in their careers. For example, several managers noted that some PS struggled with oversharing, inappropriate sharing, or sharing in ways that overly centered their own lived experience and training and supervision could help address these concerns (see Table 4, Subtheme 1c). Additionally, some programs implemented trainings on self-disclosure that sought to ensure that peers shared relevant aspects of their lived experience in a manner that was both personally comfortable as well as effective for the client.

Due to PS’ fluid client-serving roles, managers reported that PS benefit from two types of supervision—individual and group-based (see Table 4, Subthemes 3a and 3b). Individual supervision activities, including open-door supervision whereby PS had unrestricted access to program managers, aim to provide PS with immediate feedback and support regarding client care or productivity. In contrast, group-based supervision allowed for a collaborative approach to problem solving and capacity building while also enabling PS to discuss shared concerns and for supervisors to adjust and redistribute caseloads as needed. PS received supervision and support from administrative staff and clinical staff (e.g., therapists), and in some cases, more senior and experienced PS who were able to provide mentorship to their colleagues.

## Discussion

This study characterizes how program managers of outpatient mental health clinics serving TAY in Southern California conceive of peer work and how their programs support PS as they interface with clients and colleagues as they carry out their professional duties. Managers reported that PS’ client-focused and administrative responsibilities are multifaceted and wide-ranging and may require unique skill sets. For example, PS may provide emotional support, facilitate group activities, conduct outreach and field-based services, collaborate on treatment teams, and undertake administrative tasks such as documentation of services provided. These findings are consistent with existing research which shows that peers’ responsibilities vary greatly both within and between organizations (Blash, Chan, & Chapman, 2015; Chapman, Blash, Mayer, & Spetz, 2018; Cronise, Teixeira, Rogers, & Harrington, 2016; Jones, Teague, et al., 2020; Ojeda et al., 2020; Salzer, Schwenk, & Brusilovskiy, 2010).

Several managers identified flexibility as an important characteristic of successful PS, particularly because clients’ needs may differ over time and may vary across clients. However, role ambiguity may be experienced by the PS or their colleagues. Thus, a greater breadth and depth of supervision, support and training may be needed to ensure that PS have the resources needed to be successful. This study identified diverse supervision strategies and trainings as a means of addressing professional development and workplace challenges, including building PS technical and administrative skills (e.g., time management), disclosure, and interpersonal relationships (Baggetta & Alexander, 2016). These efforts may build PS’ skills in support of their professional development and long-term career trajectories (Delman & Klodnick, 2017).

Managers also reported that PS’ supervisors can serve as a bridge between PS and program staff, helping to foster team members’ understanding of the PS role and facilitating the integration of peers into teams and team activities (e.g., meetings). These strategies are in alignment with recommendations provided by the “National Practice Guidelines for Peer Specialists and Supervisors,” which suggests that mutual respect with clearly defined boundaries, coaching, and ongoing reciprocal dialogue and collaboration throughout supervision activities are among the strategies that supervisors can employ in support of PS (National Association of Peer Supporters, 2019).

Stefancic and colleagues found that having supervisors and PS engage in ongoing discussions regarding PS roles and responsibilities can foster a mutual understanding of the program structure, services, and client goals while also reducing PS’ stressors tied to role ambiguity (Stefancic et al., 2021). Furthermore, ensuring that PS have a space and mechanism to provide the program and team members with feedback is important to PS’ feeling valued and treated as equal partners (Stefancic et al., 2021). Kuhn and colleagues found that job satisfaction was greater among PS when they felt their supervisors had a deeper understanding of their role (Kuhn, Bellinger, Stevens-Manser, & Kaufman, 2015). Taken together, these findings suggest that supervisors likely need to implement multiple strategies in order to foster trust, respect and understanding of PS’ roles and their contributions to the program among all staff members and the critical role that PS play in TAY clients’ recovery process; such efforts may help create more cohesive teams and benefit client care and this should be evaluated more fully.

Prior studies have documented the emotional labor associated with peer work and its impact on PS’ mental health (Mancini & Lawson, 2009; Moran, Russinova, Gidugu, & Gagne, 2013). Our study found that programs implement both structured and unstructured opportunities for PS to receive support and supervision. An open-door policy can facilitate peers’ ongoing access to supervisors or colleagues to address self-care and the development of coping skills to manage diverse client-related or work-place related stressors. Such approaches may help foster persistence and resilience to work in challenging situations as well as confidence to carry out the PS role (Delman & Klodnick, 2017) and potentially may reduce feelings of burnout as evidenced among PS with lower levels of self-efficacy (Park, Chang, Mueller, Resnick, & Eisen, 2016).

Prior studies have found that PS may need additional support to address cultural dimensions of client care (Delman & Klodnick, 2017). Nationally, there are concerted efforts designed to support inclusivity with the goal of reducing disparities in service utilization and engagement among racial/ethnic subgroups and sexual and gender minorities (SGM) (National Institutes of Health: National Institute on Minority Health and Health Disparities, 2021; National Institutes of Health: Sexual and Gender Minority Research Office, 2021). This study found that programs serving TAY living with serious mental illness sought to address cultural dimensions of care by engaging PS from diverse racial/ethnic and linguistic subgroups that are reflective of the community they serve. Additionally, program managers were mindful of the need to engage in ongoing cultural competency trainings, particularly to foster inclusivity of SGM TAY. For example, programs developed or strengthened collaborations with community agencies with expertise in SGM well-being and services to build PS and other staff members’ knowledge of current terminology. Programs also actively sought to demonstrate the agency’s allyship with the SGM community through creating welcoming programmatic spaces and developing events for SGM TAY. Broader assessment of these approaches is needed to understand how these efforts contribute to SGM TAYs’ uptake of services.

### Limitations

This study focused on providing an organizational perspective on PS training and supervision; PS perspectives were also collected as part of the larger study and findings of that analysis will be published separately due to the expansiveness of the data. Our study relied on semi-structured interviews with program managers which may be impacted by recall bias. The study did not implement member checking strategies and this may be a valuable approach to use in future studies to ensure comprehensiveness of the data and that interpretation of interviews was consistent with the participants’ intent. Due to the onset of the COVID-19 pandemic during data analysis, engagement of participants would have been difficult to California’s strict policies which led to a long-term implementation of remote work and quarantine period. The study did not collect the demographic data of supervisors and this should be included in future studies; such data may facilitate exploration of racial/ethnic concordance of PS and supervisors in settings serving TAY. The study was conducted in two large urban communities in Southern California which may limit generalizability of findings; future studies should expand to other contexts (e.g., rural or suburban communities). Interviews were conducted immediately prior to the COVID-19 pandemic and the approaches described here may have been subsequently adapted to address emergent public health directives; further research is needed to ascertain how the pandemic impacted organizational support of PS as well as service delivery for TAY clients. California lacked standardized guidelines for PS at the time of the study, likely resulting in greater variation of the PS role and supervision activities, however, that is expected to change with finalization of the peer certification law which will be in effect in 2022 (California Department of Health Care Services, 2021). The study did not collect data on whether the supervisors believed they needed further training to better support Peer Specialists—these factors should also be explored in a further study.

## Conclusions

This study provides a recent view of program managers’ perspectives on the role of PS within mental health service programs that serve TAY living with serious mental illness. Findings demonstrate that program managers appreciate and understand the value of peer support within mental health services that seek to engage diverse TAY clients, as well as the complexity of the PS role. Consequently, managers responded with varied strategies to support PS so that they may be personally and professionally successful. Results from this study may inform the field on how to improve the implementation and sustainment of high-quality peer support, while also providing insight regarding the long-term development of PS and protocols to support them and their careers; these are neglected areas of focus. Longitudinal research is needed to assess the impact of such organizational efforts on PS’ job satisfaction and productivity, career trajectories, as well as TAY clients’ engagement with services, particularly among racial/ethnic minority and sexual and gender minority TAY clients.

## References

[CR1] Baggetta, P., & Alexander, P. A. (2016). Conceptualization and operationalization of executive function. *Mind, Brain, and Education, 10*(1), 10–33.

[CR2] Blash, L., Chan, K., & Chapman, S. (2015). *The Peer Provider Workforce in Behavioral Health: A Landscape Analysis*: University of California, San Francisco.

[CR4] California Department of Health Care Services. (2021). Peer Support Services. Retrieved March 3, 2021, from https://www.dhcs.ca.gov/services/Pages/Peer-Support-Services.aspx

[CR3] Bonnie, R. J. (2014). *Investing in the health and well-being of young adults*. Washington, DC: National Academies Press. 25855847

[CR5] Chapman, S. A., Blash, L. K., Mayer, K., & Spetz, J. (2018). Emerging Roles for Peer Providers in Mental Health and Substance Use Disorders. *American Journal of Preventive Medicine, 54*(6 Suppl 3), S267-S274. 10.1016/j.amepre.2018.02.019 29779551

[CR6] Chinman, M., George, P., Dougherty, R. H., Daniels, A. S., Ghose, S. S., Swift, A., et al. (2014). Peer support services for individuals with serious mental illnesses: assessing the evidence. *Psychiatr Serv, 65*(4), 429–441. 10.1176/appi.ps.201300244 24549400

[CR7] Clarke, V., & Braun, V. (2014). Thematic analysis *Encyclopedia of critical psychology* (pp. 1947–1952): Springer.

[CR8] Creswell, J. W., & Hirose, M. (2019). Mixed methods and survey research in family medicine and community health. *Family Medicine and Community Health, 7*(2), e000086. 10.1136/fmch-2018-000086 PMC6910743 32148709

[CR9] Cronise, R., Teixeira, C., Rogers, E. S., & Harrington, S. (2016). The peer support workforce: Results of a national survey (pp. 211–221): Educational Publishing Foundation. 10.1037/prj0000222 27618458

[CR10] Cusick, G. R., Havlicek, J. R., & Courtney, M. E. (2012). Risk for arrest: The role of social bonds in protecting foster youth making the transition to adulthood. *The American Journal of Orthopsychiatry, 82*(1), 19–31. 10.1111/j.1939-0025.2011.01136.x PMC3470487 22239390

[CR11] Davis, M. (2003). Addressing the needs of youth in transition to adulthood. *Administration and Policy in Mental Health, 30*(6), 495–509. 10.1023/a:1025027117827 13677456

[CR12] Delman, J., & Klodnick, V. V. (2017). Factors supporting the employment of young adult peer providers: Perspectives of peers and supervisors. *Community mental health journal, 53*(7), 811–822. 10.1007/s10597-016-0059-6 27770306

[CR13] Hegelstad, W. t. V., Larsen, T. K., Auestad, B., Evensen, J., Haahr, U., Joa, I., et al. (2012). Long-term follow-up of the TIPS early detection in psychosis study: effects on 10-year outcome. *American Journal of Psychiatry, 169*(4), 374–380. 10.1176/appi.ajp.2011.11030459 22407080

[CR14] Jivanjee, P., Grover, L., Thorp, K., Masselli, B., Bergan, J., & Brennan, E. M. (2020). Training needs of peer and non-peer transition service providers: Results of a national survey. *The journal of behavioral health services & research, 47*(1), 4–20. 10.1007/s11414-019-09667-3 31240441

[CR15] Jones, N., Kosyluk, K., Gius, B., Wolf, J., & Rosen, C. (2020). Investigating the mobility of the peer specialist workforce in the United States: Findings from a national survey. *Psychiatric rehabilitation journal, 43*(3), 179. 10.1037/prj0000395 31789547

[CR16] Jones, N., Teague, G. B., Wolf, J., & Rosen, C. (2020). Organizational climate and support among peer specialists working in peer-run, hybrid and conventional mental health settings. *Administration and Policy in Mental Health and Mental Health Services Research, 47*(1), 150–167. 10.1007/s10488-019-00980-9 31564032

[CR17] Kane, J. M., Robinson, D. G., Schooler, N. R., Mueser, K. T., Penn, D. L., Rosenheck, R. A., et al. (2016). Comprehensive versus usual community care for first-episode psychosis: 2-year outcomes from the NIMH RAISE early treatment program. *American Journal of Psychiatry, 173*(4), 362–372. 10.1176/appi.ajp.2015.15050632 PMC4981493 26481174

[CR18] Kuhn, W., Bellinger, J., Stevens-Manser, S., & Kaufman, L. (2015). Integration of peer specialists working in mental health service settings. *Community Ment Health J, 51*(4), 453–458. 10.1007/s10597-015-9841-0 25672842

[CR19] Landis, J. R., & Koch, G. G. (1977). The measurement of observer agreement for categorical data. *biometrics*, 159–174. 843571

[CR20] Maguire, M., & Delahunt, B. (2017). Doing a Thematic Analysis: A Practical, Step-by-Step Guide for Learning and Teaching Scholars. *All Ireland Journal of Higher Education, 9*(3).

[CR21] Mahlke, C. I., Krämer, U. M., Becker, T., & Bock, T. (2014). Peer support in mental health services. *Curr Opin Psychiatry, 27*(4), 276–281. 10.1097/YCO.0000000000000074 24852058

[CR22] Mancini, M. A., & Lawson, H. A. (2009). Facilitating Positive Emotional Labor in Peer-Providers of Mental Health Services. *Administration in Social Work, 33*(1), 3–22.

[CR23] Moran, G. S., Russinova, Z., Gidugu, V., & Gagne, C. (2013). Challenges experienced by paid peer providers in mental health recovery: a qualitative study. *Community Ment Health J, 49*(3), 281–291. 10.1007/s10597-012-9541-y 23117937

[CR24] Munson, M. R., Jaccard, J., Scott Jr, L. D., Moore, K. L., Narendorf, S. C., Cole, A. R., et al. (2021). Outcomes of a Metaintervention to Improve Treatment Engagement Among Young Adults With Serious Mental Illnesses: Application of a Pilot Randomized Explanatory Design. *Journal of Adolescent Health*. 10.1016/j.jadohealth.2021.04.023 PMC8545849 34099390

[CR25] Munson, M. R., & McMillen, J. C. (2009). Natural Mentoring and Psychosocial Outcomes among Older Youth Transitioning From Foster Care. *Children and youth services review, 31*(1), 104–111. 10.1016/j.childyouth.2008.06.003 PMC2630481 20046218

[CR26] National Association of Peer Supporters. (2019). *National Practice Guidelines for Peer Specialists and Supervisors*. Washington, DC: National Association of Peer Supporters.

[CR27] National Institutes of Health: National Institute on Minority Health and Health Disparities. (2021). *Minority Health and Health Disparities Strategic Plan 2021–2025*. Washington, DC: National Institutes of Health.

[CR28] National Institutes of Health: Sexual and Gender Minority Research Office. (2021). *Strategic Plan to Advance Research on the Health and Well-being of Sexual and Gender Minorities Fiscal Years 2021–2025*. Washington, DC: The National Insitutes of Health Sexual and Gender Minority Research Ofice.

[CR29] O’Connor, C., & Joffe, H. (2020). Intercoder reliability in qualitative research: debates and practical guidelines. *International Journal of Qualitative Methods, 19*, 1609406919899220.

[CR30] Ojeda, V. D., Hiller, S. P., Hurst, S., Jones, N., McMenamin, S., Burgdorf, J., et al. (2016). Implementation of Age-Specific Services for Transition-Age Youths in California. *Psychiatric Services, 67*(9), 970–976. 10.1176/appi.ps.201500084 27133720

[CR31] Ojeda, V. D., Jones, N., Munson, M. R., Berliant, E., & Gilmer, T. P. (2020). Roles of peer specialists and use of mental health services among youth with serious mental illness. [https://doi.org/10.1111/eip.13036]. *Early Intervention in Psychiatry, n/a*(n/a). 10.1111/eip.13036 PMC9305632 32888260

[CR32] Palinkas, L. A., Horwitz, S. M., Green, C. A., Wisdom, J. P., Duan, N., & Hoagwood, K. (2015). Purposeful Sampling for Qualitative Data Collection and Analysis in Mixed Method Implementation Research. *Administration and Policy in Mental Health and Mental Health Services Research, 42*(5), 533–544. 10.1007/s10488-013-0528-y PMC4012002 24193818

[CR33] Park, S. G., Chang, B.-H., Mueller, L., Resnick, S. G., & Eisen, S. V. (2016). Predictors of Employment Burnout Among VHA Peer Support Specialists. *Psychiatric Services, 67*(10), 1109–1115. 10.1176/appi.ps.201500239 27247169

[CR34] Roberts, K., Dowell, A., & Nie, J.-B. (2019). Attempting rigour and replicability in thematic analysis of qualitative research data; a case study of codebook development. *BMC medical research methodology, 19*(1), 1–8. 10.1186/s12874-019-0707-y PMC6437927 30922220

[CR35] Salzer, M. S., Schwenk, E., & Brusilovskiy, E. (2010). Certified peer specialist roles and activities: results from a national survey. *Psychiatr Serv, 61*(5), 520–523. 10.1176/ps.2010.61.5.520 20439376

[CR36] Shalaby, R. A. H., & Agyapong, V. I. O. (2020). Peer Support in Mental Health: Literature Review. *JMIR mental health, 7*(6), e15572-e15572. 10.2196/15572 PMC7312261 32357127

[CR37] Simmons, M. B., Grace, D., Fava, N. J., Coates, D., Dimopoulos-Bick, T., Batchelor, S., et al. (2020). The Experiences of Youth Mental Health Peer Workers over Time: A Qualitative Study with Longitudinal Analysis. *Community Mental Health Journal, 56*(5), 906–914. 10.1007/s10597-020-00554-2 31970578

[CR38] Stefancic, A., Bochicchio, L., Tuda, D., Harris, Y., DeSomma, K., & Cabassa, L. J. (2021). Strategies and Lessons Learned for Supporting and Supervising Peer Specialists. *Psychiatric Services, 72*(5), 606–609. 10.1176/appi.ps.202000515 PMC8656242 33657843

[CR39] VERBI Software. (20219). MAXQDA 2020. Berlin, Germany: VERBI Software

[CR40] Vostanis, P. (2005). Patients as parents and young people approaching adulthood: How should we manage the interface between mental health services for young people and adults? *Current Opinion in Psychiatry, 18*(4), 449–454. 10.1097/01.yco.0000172067.32014.91 16639141

